# Privacy Protection for Open Sharing of Psychiatric and Behavioral Research Data: Ethical Considerations and Recommendations

**DOI:** 10.31083/AP38759

**Published:** 2025-02-28

**Authors:** Yundi Zhang, Siyuan Fan, Hui Hui, Ning Zhang, Jing Li, Liping Liao, Chaofu Ke, Dan Zhang, Shihong Su, Zhiqiang Song, Yu Zhang, Qian Du, Long Liu, Lan Wang, Lijie Yang, Jia Li, Li Xu, Shiqi Xiao, Lei Shi, Xuman Xiao, Wenzhao Wang, Niuniu Sun, Qilian He, Ran Hao, Ju Wu, Zhiqiang Tian, Yanting Lou, Qiang Yao, Wai-kit Ming, Feng Jiang, Xiaoming Zhou, Mingxu Wang, Xinying Sun, Yibo Wu, on behalf of Behavioral Epidemiology Education Working Commission of CMEA

**Affiliations:** ^1^School of Journalism, Fudan University, 200437 Shanghai, China; ^2^Yanjing Medical College, Capital Medical University, 100054 Beijing, China; ^3^Department of Coronary Heart Disease, Central Hospital of Dalian University of Technology (Dalian Municipal Central Hospital), 116033 Dalian, Liaoning, China; ^4^School of Public Health and the Second Affiliated Hospital, Zhejiang University School of Medicine, 310058 Hangzhou, Zhejiang, China; ^5^Department of Maternal Group Health, Tangshan Maternal and Children Health Hospital, 063000 Tangshan, Hebei, China; ^6^Department of Respiratory and Critical Care Medicine, Zigong First People’s Hospital, 643000 Zigong, Sichuan, China; ^7^School of Public Health, Suzhou Medical College of Soochow University, 215123 Suzhou, Jiangsu, China; ^8^School of Basic Medical Sciences, Shandong University, 250014 Jinan, Shandong, China; ^9^Department of Respiratory and Critical Care Medicine, The First Affiliated Hospital of Anhui Medical University, 230022 Hefei, Anhui, China; ^10^Department of Gastroenterology, Peking University Third Hospital, 100191 Beijing, China; ^11^National Institute for Viral Disease Control and Prevention, Chinese Center for Diseases Control and Prevention, 102206 Beijing, China; ^12^Department of Pharmacy, The Third Affiliated Hospital of Chongqing Medical University, 401120 Chongqing, China; ^13^School of Health Care, Chongqing Preschool Education College, 404047 Chongqing, China; ^14^School of Nursing, Tianjin Medical University, 300070 Tianjin, China; ^15^Department of Pharmacy, The Sixth Affiliated Hospital of Harbin Medical University, 150023 Harbin, Heilongjiang, China; ^16^Department of Scientific Research and Education, Changchun Sixth Hospital, 130052 Changchun, Jilin, China; ^17^Department of Publicity and Promotion, The Changchun Municipal Health Education Center, 130022 Changchun, Jilin, China; ^18^Department of Inpatient, Shengjing Hospital of China Medical University, 110004 Shenyang, Liaoning, China; ^19^School of Health Management, Guangzhou Medical University, 511436 Guangzhou, Guangdong, China; ^20^Department of Psychiatry, The Third People’s Hospital of Foshan City, 528041 Foshan, Guangdong, China; ^21^Department of Orthopedics, Qilu Hospital of Shandong University, 250012 Jinan, Shandong, China; ^22^School of Nursing, Henan University of Science and Technology, 471023 Luoyang, Henan, China; ^23^School of Nursing, Dali University, 671003 Dali, Yunnan, China; ^24^Nursing School, Hebei Medical University, 050017 Shijiazhuang, Hebei, China; ^25^Department of Organization, Shanxi Bethune Hospital, 030032 Taiyuan, Shanxi, China; ^26^Department of Urology, Chaohu Hospital of Anhui Medical University, 238000 Chaohu, Anhui, China; ^27^School of Political Science and Public Administration, Wuhan University, 430072 Wuhan, Hubei, China; ^28^Department of Infectious Diseases and Public Health, Jockey Club College of Veterinary Medicine and Life Sciences, City University of Hong Kong, Hong Kong, China; ^29^School of International and Public Affairs, Shanghai Jiao Tong University, 200240 Shanghai, China; ^30^Department of Research, Shandong Provincial Hospital Affiliated to Shandong First Medical University, 250021 Jinan, Shandong, China; ^31^School of Public Health, Xi’an Jiaotong University, 710049 Xi’an, Shaanxi, China; ^32^School of Public Health, Peking University, 100871 Beijing, China

**Keywords:** psychiatric and behavioral research data, privacy protection, code of ethics

## Abstract

Data sharing within psychiatric and behavioral research represents a novel application of ethical principles in practice; however, it suffers from a dearth of practical experience and established ethical norms. In this study, we comprehensively examined the ethical considerations surrounding the acquisition, management, sharing, and utilization of such data. We graded sensitive data and suggest ethical standards for privacy protection based on varying levels of data sensitivity. The objective of this study is to foster orderly and standardized open sharing of psychiatric and behavioral research data, thereby advancing the development and progress of related academic disciplines in China. This Chinese expert consensus has been registered on the International Guide Registration platform (Registration Number: PREPARE-2024CN412).

## Main Points

1. The study emphasizes the need for establishing ethical norms for the acquisition, management, sharing, and use of psychiatric and behavioral research data, particularly in the context of open privacy protection.

2. We propose a tiered classification system for data sensitivity, dividing data into highly sensitive, moderately sensitive, and low-sensitive categories, each requiring different levels of privacy protection measures.

3. The importance of data privacy is highlighted, with recommendations for strict data encryption, access control, and anonymization to prevent unauthorized access and misuse, especially for highly sensitive data.

4. The study identifies ethical challenges at both the data collector and user levels, offering recommendations such as developing strict data management policies, enhancing data ethics education, and implementing review and accountability mechanisms.

5. The research aims to promote the orderly and standardized sharing of psychiatric and behavioral data, which is crucial for advancing academic exchanges, scientific research cooperation, and the overall progress of related disciplines, including mental health investigations and promotion.

## 1. Status and Ethical Research Basis of Open Privacy Protection for Psychiatric and Behavioral Research Data

### 1.1 Importance of Data Privacy

As society progresses and education improves, there is a growing recognition of the significance of mental well-being [1]. Consequently, mental health data resources have become increasingly important. Psychiatric and behavioral research data frequently contain private information about individuals. Should these data be leaked or misused, it could severely compromise individuals’ rights and interests, and potentially even impair their mental health [2]. Furthermore, inadequate protection of research data can significantly erode public trust in scientific research, undermining its sustainability. Such issues could ultimately impede research advancements and the enhancement of public mental health. Additionally, breaches in data privacy can harm specific demographic groups from a policy-making standpoint and pose substantial legal and economic risks to organizations responsible for collecting, storing, and utilizing the data.

### 1.2 Characteristics of Psychological and Behavioral Data

Psychological and behavioral data possess several distinct characteristics that influence their collection and interpretation:

**Subjectivity**: Psychological and behavioral data are inherently subjective, as they are shaped by an individual’s personal feelings and cognitions [3]. Data collection often relies on methods such as questionnaires and interviews, which can be influenced by the investigator’s own perceptions, thus introducing subjectivity into the data.

Psychological and behavioral data are diverse. Individuals’ psychology and behavior are influenced by various factors, such as cultural background, social environment, personal experience, and so on.

**Diversity**: These data types are also highly diverse, reflecting the myriad of factors that influence an individual’s psychology and behavior, including cultural background, social environment, and personal experiences. This diversity underscores the complexity of psychological and behavioral research and the need for nuanced analysis [4].

**Sensitivity**: Lastly, psychological and behavioral data are often sensitive, containing personal information about individuals, such as personality traits and mental health status. The handling of such data requires careful attention to privacy and ethical considerations to protect individual privacy [5].

### 1.3 Current State of Psychiatric and Behavioral Research Data Openness

The Basic Medical Care and Health Promotion Law of the People’s Republic of China regulates the provision of basic medical care and health services, health promotion activities, and the supervision and management of related activities in China, emphasizing the principle of focusing on people’s health and the implementation of the “Healthy China” strategy [6].

With the advent of the digital era, digital health has become a key area in building a digital China. China is likely to strengthen and improve digital healthcare legislation and develop and improve systems for building, opening, sharing, and trading medical data. China is improving health laws and regulations, further reforming the medical and healthcare systems, and establishing a system that prioritizes the development of people’s health and a system for evaluating and assessing the impact of health [7].

The current state of psychiatric and behavioral research data openness in China is evolving. The Chinese psychological community recognizes the value of data sharing and is increasingly encouraging researchers to make their findings publicly accessible [8]. This gradual progress towards data openness is supported by several psychiatric and behavioral data-sharing platforms, which offer convenient data access and sharing channels, significantly advancing research in related domains.

**Fig. 1.**
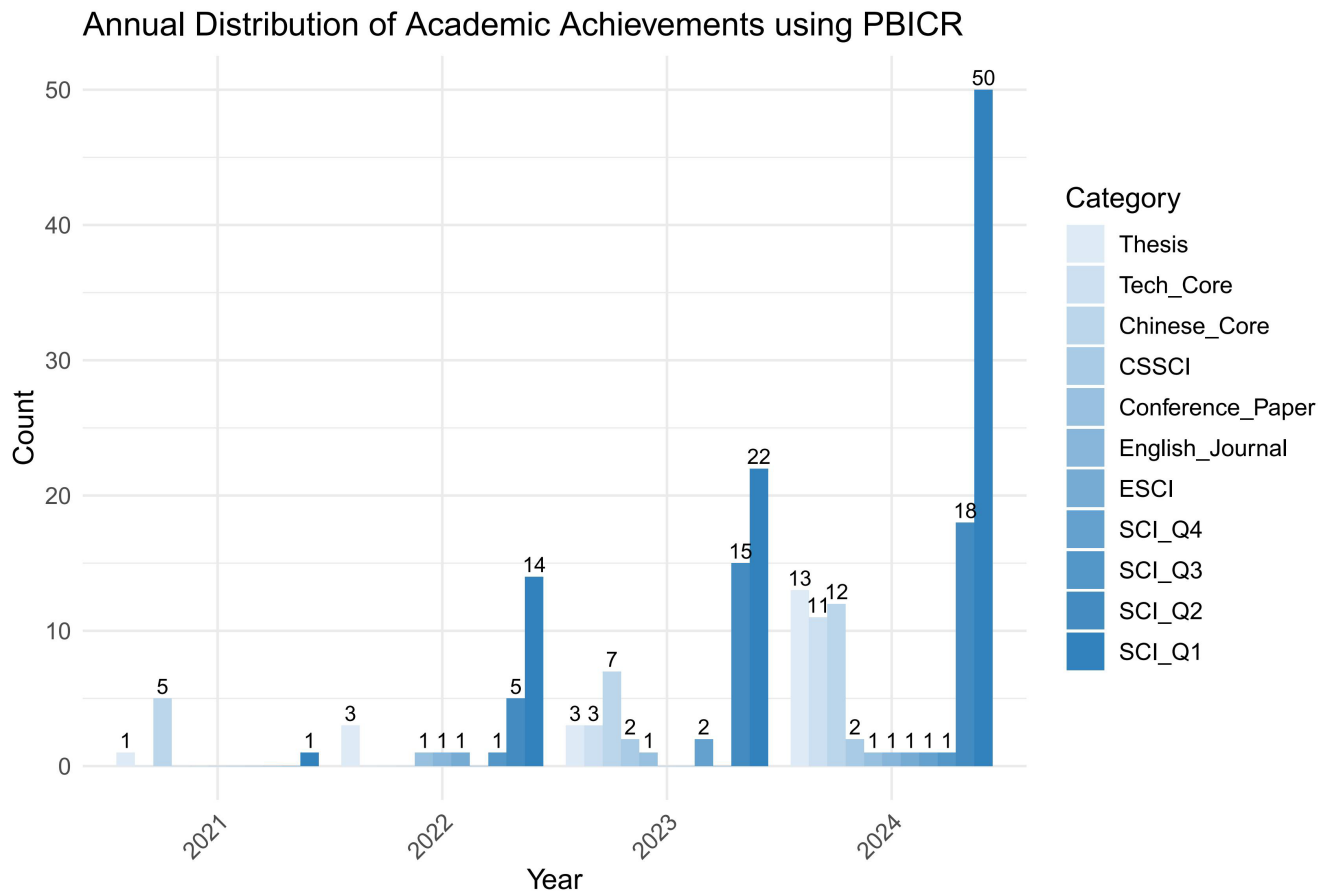
**Statistics on the number of papers published using Psychology and Behavior Investigation of Chinese Residents (PBICR) data**. CSSCI, Chinese Social Sciences Citation Index; ESCI, Emerging Sources Citations Index; SCI, Science Citation Index.

The Psychology and Behavior Investigation of Chinese Residents (PBICR) exemplifies this positive trend by making its data accessible to a broad spectrum of researchers, thereby fostering data sharing and application, as well as providing rich resources for in-depth studies across various disciplines. As of August, 2024, scholars have utilized PBICR data to publish 199 papers, including 131 in SCI/SSCI journals, and to submit 538 diverse research hypotheses. Furthermore, 20 Doctoral and Master’s students have employed PBICR data from 2020 to 2024 as the cornerstone of their thesis [9]. Fig. 1 shows the number of papers published using PBICR data from 2021–2024.

Beyond PBICR, China hosts other significant databases and research projects, such as the China Health and Retirement Longitudinal Study (CHARLS), which collects longitudinal data on health, economics, social aspects, and psychiatry among China’s middle-aged and elderly populations, bolstering research in these areas. Other initiatives include the China Family Panel Studies (CFPS), China Labor Dynamics Survey (CLDS), China Learning to Age Socially Tracking Survey (CLASS), China General Social Survey (CGSS), and Chinese Longitudinal Healthy Longevity Survey (CLHLS). Open data usage is on the rise, with CHARLS reporting a 25% increase in participating organizations and CFPS a 40% increase in data downloads over the past 2 years [10]. Other initiatives include the China Family Panel Studies (CFPS), China Labor Dynamics Survey (CLDS), China Learning to Age Socially Tracking Survey (CLASS), China General Social Survey (CGSS), and Chinese Longitudinal Healthy Longevity Survey (CLHLS). Open data usage is on the rise, with CHARLS reporting a 25% increase in participating organizations and CFPS a 40% increase in data downloads over the past 2 years [11]. These platforms are crucial for convenient access and facilitate research progress in related fields.

Globally, numerous databases and research projects are dedicated to mental and behavioral health research. Examples include the National Institute of Mental Health Data Archive (NDA), the Substance Abuse and Mental Health Services Administration (SAMHSA), and the Mental Health Data Hub (NHS England). Collectively, these form a robust global knowledge network aimed at deepening our understanding and management of mental and behavioral health.

### 1.4 Research Basis

The research basis for the present study is grounded in a dual framework of normative regulations and expert consensus. On one hand, it draws upon international and domestic legal systems, consent models, normative documents, and privacy protection guidelines [12]. On the other hand, it incorporates insights from expert groups specifically convened to shape this ethical consensus.

In China, a comprehensive set of legal protections and processes has been established to address privacy violations of psychological and behavioral data. According to the Civil Code of the People’s Republic of China and the Law on the Protection of Personal Information, medical institutions and their medical staff have a legal obligation to protect patients’ privacy and personal information. In the event of a privacy violation, the medical institution must take immediate and effective countermeasures and report the incident to the healthcare administration, as well as initiate an internal investigation to assess the impact of the leakage and prevent further leakage. Illegal disclosure of personal information may result in administrative penalties, including warnings and fines, and may even lead to suspension or revocation of the license to practice. In serious cases, those responsible may face criminal liability under the Criminal Law of the People’s Republic of China. Patients also have the right to defend their rights and interests through legal means, including filing civil lawsuits for compensation for moral damages. In addition, China has emphasized legal requirements for the handling of personal information, particularly for sensitive information such as medical and health data, which requires the individual’s separate consent, and has strictly regulated the handling of personal information in violation of the law. Together, these series of measures constitute China’s infrastructure for psychological and behavioral data privacy protection, designed to ensure that individual privacy is protected, data security is maintained, and legal remedies are available to victims [13]. Together, these series of measures constitute China’s infrastructure for psychological and behavioral data privacy protection, designed to ensure that individual privacy is protected, data security is maintained, and legal remedies are available to victims.

Furthermore, within China, medical associations and similar organizations are tasked with establishing industry standards, providing professional training, and fostering adherence to medical ethics. While they may investigate ethical issues in medical practice, they typically do not intervene in privacy invasion cases, which are primarily addressed by legal entities or data protection agencies. Ethics committees (IRBs) in China play a pivotal role in reviewing research proposals involving human subjects, ensuring ethical and regulatory compliance. Their responsibilities encompass assessing the scientific and ethical aspects of research proposals, safeguarding participant rights, and supervising research conduct. Psychiatry and behavioral science research projects are subject to the same rigorous ethical review process, evaluating the research’s purpose, data collection methods, potential risks, and mitigation strategies. They assess the scientific validity of the research protocol, the risk-to-benefit ratio for the subjects, the standardization of informed consent, respect for the rights of the subjects, and adherence to the code of research integrity. All clinical research projects must be reviewed and approved by the Ethics Review Committee before they are conducted, and further follow-up reviews may be conducted during the course of the study to monitor the execution of the study. The ethical review and related personnel shall abide by the Constitution, laws, and relevant regulations of the People’s Republic of China to ensure that the rights of research participants are respected and protected [14].

Meanwhile, there is a special ethics committee responsible for evaluating and approving research projects in the field of psychiatry and behavioral sciences from an ethical perspective. According to the Measures for Ethical Review of Biomedical Research Involving Human Beings issued by the National Health and Wellness Commission, these committees must be set up in medical institutions at or above the second level, health institutions at or above the municipal level of districts, institutions of higher education and scientific research institutes, etc., in order to ensure that all biomedical research projects involving human beings, including psychiatry and behavioral sciences research, undergo strict ethical review. Ethics committees conduct reviews based on a series of criteria, including the scientific validity of research protocols, fairness in the selection of subjects, the ratio of risks to benefits, the standardization of informed consent, respect for the rights of subjects, and adherence to scientific integrity. In addition, the Ethics Committee is responsible for following up and reviewing approved research projects to ensure that the implementation of the research complies with ethical standards. For research studies in which prior informed consent from subjects cannot be obtained under special circumstances, the Ethics Committee has the authority to approve ex post facto informed consent or waive the need to seek informed consent again. Together, these measures safeguard the rights and interests of subjects with mental disorders, reflecting China’s progress in the ethical review of psychiatry and the importance it attaches to the protection of subjects [15].

In response to privacy violations, China has implemented comprehensive laws and regulations, including data breach notification, data protection impact assessments, and legal sanctions for infractions. The Civil Code, Personal Information Protection Law, and Cybersecurity Law articulate the principles and requirements for safeguarding personal information and privacy, emphasizing legality, legitimacy, and necessity in the collection, storage, and use of such data [9]. The Data Security Law mandates that data processors implement technical and managerial measures to secure personal data, while Criminal Law has established the “crime of infringing on citizens’ personal information”, defining criminal liability for severe violations [16, 17].

**Fig. 2.**
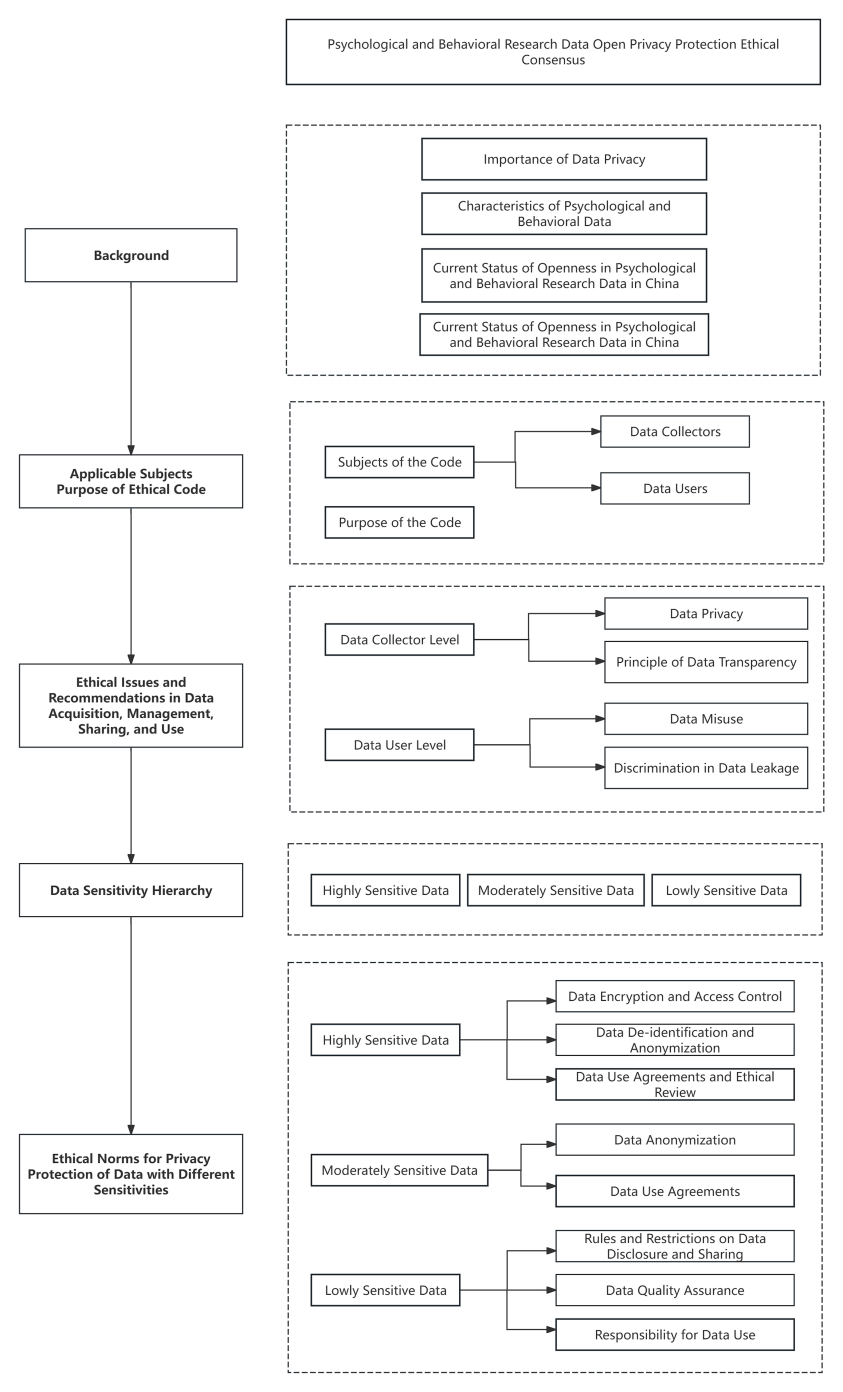
**Technology roadmap**.

Internationally, legal instruments such as the European Union’s General Data Protection Regulation (GDPR) [18] and the US Health Insurance Portability and Accountability Act (HIPAA) [19] provide detailed provisions on personal data protection, requiring consent and security measures from data processors. The GDPR is a regulation (not a directive) under EU law that provides a strong framework for data protection and privacy in the EU and EEA. HIPAA, on the other hand, provides specific guidelines for protecting sensitive patient health information in the United States. Together, these regulations provide a solid foundation for discussing data privacy and ethical considerations in database management [20]. These serve as valuable references for formulating the Ethical Consensus on Open Privacy Protection for Psychological and Behavioral Research Data. Fig. 2 outlines the technology roadmap for the present study.

## 2. The Applicable Object and Purpose of the Ethical Code of Privacy Protection

### 2.1 Norms Applicable To the Object

Ethical norms for open data and privacy protection in psychiatric and behavioral research encompass a range of stakeholders, including both data collectors and data users.

Data Collectors: This group comprises a variety of roles such as researchers from various disciplines (psychologists, social scientists, and other relevant experts), research participants (individuals who voluntarily engage in the study), ethical review boards (tasked with ensuring that research protocols meet ethical criteria), data analysts (specialized in team-based or individual data analysis), technical support staff (responsible for the accurate collection and secure storage of data, especially when specialized techniques or software are used), funding and regulatory bodies, collaborating research institutions (in cases of cross-institutional partnerships), legal advisors, publishers, and academic journals.

Data Users: This category includes a broad spectrum of individuals and entities such as principal investigators and their teams, other scientists and academics (who may utilize the data for their research, especially when it is openly shared), medical and clinical professionals, students at various levels of education, ethical review boards (assessing compliance with ethical standards), policymakers and public health officials (requiring data for developing public policies or health strategies), private sector researchers and corporations (for product design, marketing strategies, or consumer behavior research), news media and journalists, international organizations and multinational institutions (involved in cross-border research or global health initiatives), and legal professionals.

### 2.2 Purpose of the Code

The primary aim of this ethical code is to guide scientific research in a manner that protects the personal and legitimate rights of data subjects. It establishes principles and standards for data openness and sharing, particularly within the realms of genetics and neuroscience [21, 22], to mitigate the risks of data misuse and leakage. By doing so, the code enhances the standardization of data management. Furthermore, it encourages the transparent and regulated sharing of psychiatric and behavioral research data, which in turn fosters academic discourse and scientific collaboration. This ethical framework is designed to advance the field, support public health initiatives, and align with the objectives of the Healthy China 2030 plan.

## 3. Ethical Issues and Recommendations in Data Acquisition, Management, Sharing, and Use

### 3.1 Data Collector Level

#### 3.1.1 Data Privacy and its Implications

Collected data may encompass personally identifiable information (PII), and unauthorized use of such information could lead to the disclosure of individuals’ identities [23]. The unauthorized collection of behavioral and medical privacy data can significantly harm both individuals and society. To address data privacy infringement and its repercussions, the present study recommends the following measures: Data collectors should strive to minimize data collection, ensuring that only essential information is obtained, and should transparently communicate the purpose of data collection to the subjects. It is expected that data collectors will provide a comprehensive explanation of their procedures, enabling the evaluation of data quality, integrity, and the reproducibility of findings. Documentation of collected data should include details of the precautions taken to ensure data accuracy. For the collection and processing of sensitive information, detailed disclosure to the individuals concerned is mandatory, and consent must be obtained before proceeding. Post-collection, advanced data security technologies and encryption should be employed to safeguard against unauthorized access, leakage, or misuse of sensitive data. These measures are crucial for maintaining data integrity and confidentiality during transmission and storage [24].

To address data privacy infringement and its consequences, we recommend the following measures:

(1) Data collectors should aim to collect the minimum necessary data and obtain only essential information, while clearly informing individuals about the purpose of data collection, ensuring it aligns with the study’s objectives.

(2) Data collectors are expected to provide a comprehensive explanation of their procedures, enabling the evaluation of data quality, integrity, and the reproducibility of findings. Documentation should detail the precautions taken to ensure data accuracy [25].

(3) The collection and processing of sensitive information must be thoroughly communicated to the individuals involved and should only proceed with their consent. Post-collection, advanced data security technologies and encryption must be employed to prevent unauthorized access, leakage, or misuse, ensuring data integrity and confidentiality during transmission and storage.

(4) Data collection processes should establish specialized roles to classify and process data, ensuring data authenticity and effectiveness. Professionals are required for data maintenance and management, with a focus on obtaining permissions and protecting data privacy during the sharing process. It is recommended to obtain clear, voluntary, and written informed consent for the collection and application of data.

#### 3.1.2 Principle of Data Transparency

Transparency in the data collection process is crucial; respondents should be provided with clear information regarding data-related issues. The absence of informed consent can be perceived as a violation of research ethics and moral standards, potentially causing public resentment, particularly in environments emphasizing individual privacy rights. Public opinion can negatively impact researchers and institutions [26], and the scientific value of research may be questioned, reducing public trust [27]. Given the current inadequacy of data regulation, there is a recognized need and urgency for inter-departmental information sharing, collaborative mechanisms, and integrated data ethics governance [28].

To counteract the infringement of data transparency and its impact, we suggest the following:

(1) Data collectors must have clear purposes for data collection, with strict limitations on data use to those purposes, achieved through stringent research protocols and data use policies that safeguard individual privacy rights.

(2) Data collectors should deeply explore how to effectively communicate informed consent to participants, including the study’s purpose, data usage, and potential risks and benefits, using standardized informed consent forms and processes.

(3) To address the lack of data regulation, it is advised to advocate for a robust data regulatory framework, enhance inter-departmental information sharing and collaboration, and strengthen the connection between ethical data governance mechanisms.

(4) Data collectors should, when necessary, provide respondents with a clear statement on data use and privacy processes and transparently display the data handling procedures. Establishing a standardized data-sharing process, including application, review, use, and release, ensures the reasonable use of data and privacy protection.

Table 1 summarizes some of the ethical issues and recommendations at the data collector level.

**Table 1.** **Ethical issues and recommendations at the data collector level**.

Ethical issue area	Recommended measures
Data Privacy	Collect only the minimum necessary data, inform individuals clearly about the purpose of data collection, and obtain explicit consent for sensitive information.
Data Transparency	Provide detailed explanations of data collection procedures, ensure informed consent is clear, voluntary, and documented.
Data Security	Implement advanced data security technologies and encryption measures to prevent unauthorized access, leakage, or misuse of sensitive data.
Data Management	Establish strict data management policies, ensure data integrity and confidentiality during transmission and storage, and maintain professional data maintenance.

### 3.2 Data User Level

Data users play a crucial role in psychiatric and behavioral research. Not only are they responsible for interpreting and applying data to push the boundaries of scientific knowledge but they also have the important responsibility of maintaining data ethics. Ethical challenges relate to how the data are used, the impact of the data on participants’ privacy, and the interpretation of the results. In this process, data users must ensure that their work is ethical and avoid compromising individual privacy while ensuring the accuracy and reliability of the research results.

Data users are pivotal in psychiatric and behavioral research, bearing dual responsibilities: advancing scientific knowledge through data interpretation and application, and upholding data ethics. Ethical challenges at this level pertain to the usage of data, its impact on participant privacy, and the integrity of result interpretation. Data users must ensure their work adheres to ethical standards, protects individual privacy, and maintains the accuracy and reliability of research findings. Table 2 summarizes some of the ethical issues and recommendations at the data user level.

**Table 2.** **Ethical issues and recommendations at the data user level**.

Ethical issue area	Recommended measures
Data Misuse	Develop strict data management policies, enhance data ethics education for researchers, and implement review and accountability mechanisms.
Discrimination in Data Leakage	Establish fair data access and use guidelines, strengthen ethics education and training for data users, and institute effective monitoring and evaluation mechanisms.

#### 3.2.1 Data Misuse and its Implications

Data misuse refers to the utilization of data in ways that deviate from its original collection purpose, disregard ethical guidelines, or misrepresent the data’s meaning. In the context of psychiatric and behavioral research, misuse may manifest as unauthorized access to sensitive information, employing data for unauthorized research objectives, or deriving incorrect or deceptive conclusions from the data. The repercussions of data misuse are significant, potentially infringing upon participants’ privacy, undermining the integrity and reliability of research, and resulting in flawed scientific insights and policy directives. Hence, users must possess a keen understanding of how to prevent and identify data misuse.

To combat data misuse and its ramifications, we propose the following recommendations:

(1) **Establish Rigorous Data Management Policies**: It is imperative to develop stringent policies that explicitly outline the standards for data collection, storage, use, and sharing. These policies must ensure that all data-related operations adhere to both ethical and legal standards.

(2) **Enhance Data Ethics Education for Researchers**: To elevate awareness about the significance of data protection, regular data ethics training sessions should be mandated for all researchers.

(3) **Implement Review and Accountability Mechanisms**: The enforcement of a robust system of review and accountability is essential. This involves rigorous oversight of research projects by ethical review committees and holding individuals accountable for breaches of data ethics.

#### 3.2.2 Discrimination in Data Leakage

Psychiatric and behavioral data are highly sensitive as they pertain to an individual’s inner world, emotional state, and behavioral patterns. If these data are leaked, the consequences extend beyond privacy exposure; they may also impose additional psychiatric stress and discrimination on individuals, particularly due to the social stigma associated with mental health issues. The leakage of such data can induce negative emotions like anxiety and depression in individuals, stemming from the fear of privacy invasion. This emotional distress may exacerbate existing mental health conditions, leading to graver outcomes. Furthermore, data breaches can engender social misconceptions and prejudices against mental health issues, intensifying discrimination and rejection of those with such conditions. Therefore, when safeguarding the privacy of psychiatric and behavioral data, it is imperative to consider the potential social and psychiatric impacts of data leakage and to develop more stringent protection measures.

To tackle issues of data fairness and discrimination, the study recommends the following:

(1) **Establish Fair Data Access and Use Guidelines**: Develop clear guidelines ensuring equitable access to and utilization of data by research organizations, regardless of size or type [29].

(2) **Enhance Ethics Education and Training for Data Users**: Provide regular ethics education and training to data users to heighten their awareness of data fairness and discrimination issues.

(3) **Institute Effective Monitoring and Evaluation Mechanisms**: Conduct regular reviews of data use fairness and ethics to ensure adherence to established guidelines.

In conclusion, data users are instrumental in psychiatric and behavioral science research. Key strategies to mitigate risks include the establishment of strict data management policies, enhancement of ethics education, implementation of review and accountability mechanisms, and improvement of data representation and diversity. These measures are essential to ensure the ethical and fair conduct of scientific research and to foster the healthy development of the research field.

## 4. Psychiatric and Behavioral Research Data Sensitivity Hierarchy

Psychiatric and behavioral research data must be categorized and graded based on factors such as data type, privacy sensitivity of the information, data value, and security requirements. The method of privacy protection should correspond to the data tier [30, 31]. The present study referenced the EU’s Personal Data Protection Directive and the Information Technology: Big Data Data Classification Guidelines issued by China’s Standards Commission, among other regulations, to classify psychiatric and behavioral data into three tiers: highly sensitive, moderately sensitive, and minimally sensitive [32]. Table 3 summarizes psychiatric and behavioral research data sensitivity hierarchy.

**Table 3.** **Psychiatric and behavioral research data sensitivity hierarchy**.

Sensitivity	Data types and protection measures
Highly Sensitive	Personally Identifiable Information (PII), Detailed Health Records, Precise Geographic Location Information, etc.
	- Encryption and Access Control
	- Data De-identification and Anonymization
	- Data Use Agreement and Ethical Review
Moderately Sensitive	Personal Demographics, Behavioral Observation Data, etc.
	- Data Anonymization
	- Data Quality Assurance
	- Data Use Agreements
Minimally Sensitive	Aggregate Research Data, Processed Statistical Results, etc.
	- Rules and Restrictions on Data Disclosure and Sharing
	- Data Quality Assurance
	- Responsibility for Data Use

### 4.1 Highly Sensitive Data 

Highly sensitive data pertains to information that, if disclosed, could lead to severe consequences for individuals or their families [33, 34, 35]. This category encompasses, but is not limited to:

(1) **Personally Identifiable Information (PII)**: Data such as an individual’s name, ID number, and contact details are capable of identifying a person and are susceptible to identity theft or other forms of misuse [36].

(2) **Detailed Health Records**: This includes mental health assessment results, disease diagnoses, medications, and physical examination reports. Disclosure of these data can have profound psychiatric and social repercussions for the individual [37].

(3) **Precise Geographic Location Information**: Comprising the addresses of an individual’s residence, workplace, and organizational affiliations, these data can expose an individual’s living and activity patterns, thereby heightening the risk of personal privacy infringement [38].

### 4.2 Moderately Sensitive Data

Moderately sensitive data encompasses information that contains personal details yet is less private compared with highly sensitive data. A breach of this type of data might lead to minor inconvenience or harm to individuals, but it typically does not entail severe legal or economic repercussions. The following are examples of moderately sensitive data [39, 40]:

(1) **Personal Demographics**: This includes details such as age, gender, occupation, and religious beliefs. Despite anonymization efforts, the combination of these attributes with other data could potentially re-identify an individual [39].

(2) **Behavioral Observation Data**: This pertains to participants’ performance and reaction times in experiments and studies. Such data could implicate an individual’s cognitive and emotional privacy [40].

### 4.3 Minimally Sensitive Data 

Minimally sensitive data contains minimal personal information, and breaches involving this type of data typically do not lead to substantial loss or impact for individuals. Examples of minimally sensitive data include:

(1) **Aggregate Research Data**: This includes statistical data and analysis results from research samples, which are based on aggregated information from a large number of individuals and devoid of specific personal details.

(2) **Processed Statistical Results**: Such data may include summaries, charts, and other forms of research outcomes that have been processed and summarized, eliminating specific personal information or identifiable characteristics.

While less sensitive on an individual level, Low Sensitivity Data can reflect broader trends in income, psychiatric and behavioral status, and health conditions within a regional population. This information, if mishandled, could potentially lead to discrimination.

## 5. Ethical Norms of Privacy Protection for Data of Different Sensitivities

The study referenced the Implementation Measures of the Interim Provisions on the Management of International Networking of Computer Information Networks and the Code of Ethics for Clinical and Counseling Psychology Work (Second Edition) established by the Chinese Psychological Association, proposing an ethical consensus on privacy protection across three distinct levels.

The present study draws upon the Implementation Measures of the Interim Provisions on the Management of International Networking of Computer Information Networks [41] and the Code of Ethics for Clinical and Counseling Psychology Work (Second Edition) [42] established by the Chinese Psychological Association, proposing an ethical consensus on privacy protection across three distinct levels.

The study refers to the three basic principles of the 1978 Belmont Report and argues that privacy protection in the context of open data sharing should encompass three key aspects, as shown in Table 4 [43].

**Table 4.** **Ethical principles and applications outlined in the 1978 Belmont Report**.

Ethical principle	Application in the research setting
Respect for Persons	Informed consent
	– information
	– comprehension
	– voluntariness
	• Additional protections for persons with diminished autonomy
Beneficence	• Maximizing benefits for research participants and society
	• Minimizing harm to research participants
Justice	Ensuring fair distribution of the benefits and burdens of research

First, respecting an individual’s right to privacy and autonomy is the core principle of data-privacy protection. This entails ensuring that individuals fully understand and voluntarily consent to how and for what purposes their data will be used during collection, processing, and utilization. This includes not only the lawful collection of data but also maintaining confidentiality and secure storage of data [44].

Second, adequately protecting the interests of the subjects is crucial. In data collection, the minimum necessary data should be gathered, and individuals should be clearly informed of the data collection’s purpose. For sensitive information, explicit consent must be obtained, and advanced data security technologies and encryption measures should be implemented [45].

Third, promoting the advancement of scientific research and the public interest is essential. By establishing an ethical code of privacy protection based on the openness of psychological and behavioral research data, data security can be maintained, and mental health and behavioral health research can be supported in its sustainability. This is vital for the development and progress of related disciplines and the promotion of public health [46].

### 5.1 Highly Sensitive Data

**Fig. 3.**
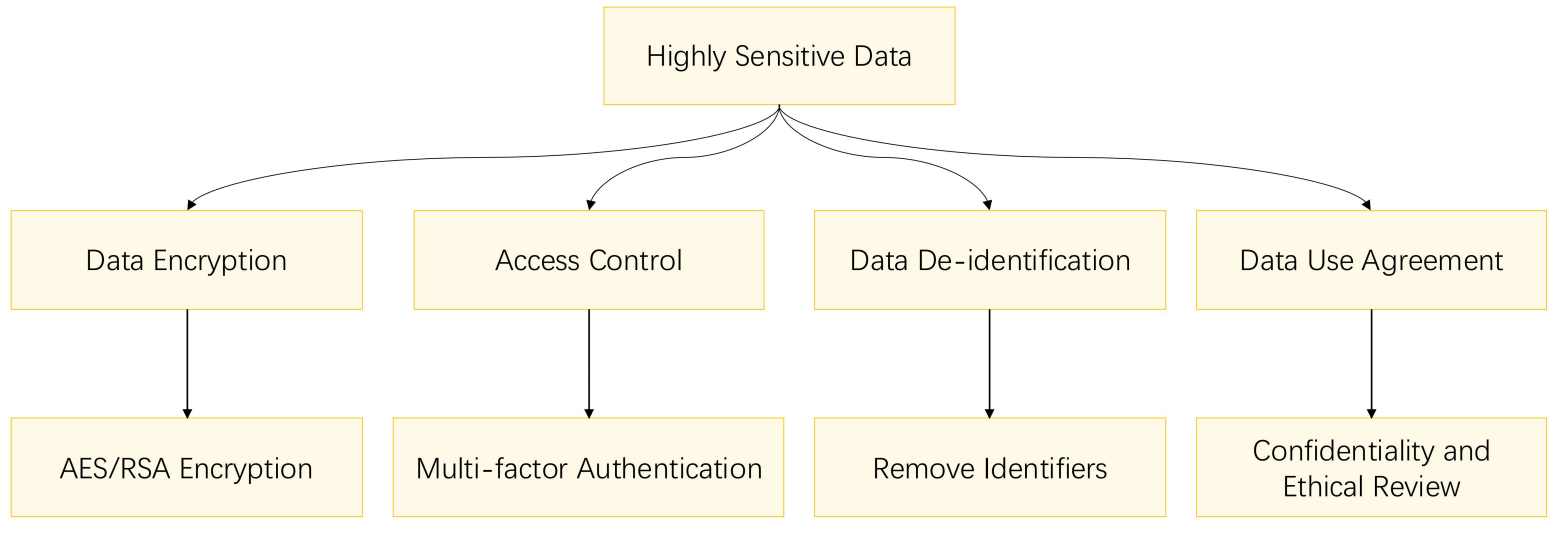
**Flowchart for highly sensitive data privacy protection**.

For highly sensitive data, the most stringent and comprehensive protection measures are imperative, as they pertain to personal privacy and rights, and their disclosure could have severe consequences for individuals. Fig. 3 outlines the privacy protection methods for highly sensitive data.

#### 5.1.1 Data Encryption and Access Control

For highly sensitive data, encryption is not only a necessary means but should be the core of multiple protection measures. All data must be encrypted using advanced encryption techniques (e.g., Advanced Encryption Standard (AES) or Rivest–Shamir–Adleman (RSA) encryption algorithms) to ensure confidentiality during storage and transmission [47].

Simultaneously, strict access control policies should be implemented. Multifactor authentication mechanisms, such as biometrics and dynamic tokens, can be introduced to ensure that only strictly verified personnel can access highly sensitive data. For example, multi-factor authentication mechanisms such as biometrics and dynamic tokens can be employed to enhance data security [48]. Access Control Encryption (ACE) is an emerging encryption paradigm that controls not only what users can read, but also what they can send [49].

#### 5.1.2 Data De-identification and Anonymization

Highly sensitive data must undergo de-identification and anonymization processes to eliminate any information that could directly or indirectly identify individuals, thereby mitigating the risk of data leakage. De-identification methods and standards involve removing personal identifiers and applying techniques such as generalization and suppression to ensure that individuals cannot be re-identified from the dataset. For instance, the ISO 27001 international standard requires organizations to establish, implement, maintain, and continuously improve an information security management system, offering practical guidance for de-identification. Furthermore, regulations such as the California Consumer Privacy Act (CCPA) and the Health Insurance Portability and Accountability Act (HIPAA) underscore the significance of de-identification and lay out specific compliance requirements [50].

Anonymization is an additional approach to safeguard individual privacy, yet it comes with limitations and challenges. Even with anonymization, it may be possible to re-identify individuals through data linking or other technical means, and excessive anonymization could diminish the data’s utility and research value. Hence, a balance must be struck between privacy protection and data utility. As suggested by Wanbil W. Lee *et al*. [51], privacy protection strategies should evolve in tandem with technological advancements, with de-identification and anonymization strategies regularly assessed and updated.

#### 5.1.3 Data Use Agreement and Ethical Review

Detailed data use agreements should be formulated, specifying data confidentiality obligations, scope of use, and restrictions on data sharing and transmission. A stricter penalty mechanism for breach of contract should be introduced to increase the cost of violating the agreement. All research projects involving highly sensitive data must undergo ethical review, which should include an assessment of the purpose of the research, the reasonableness of the data collection and processing methods, potential risks, and countermeasures, etc., to ensure that the research complies with ethical standards, laws, and regulations [52].

### 5.2 Moderately Sensitive Data

**Fig. 4.**
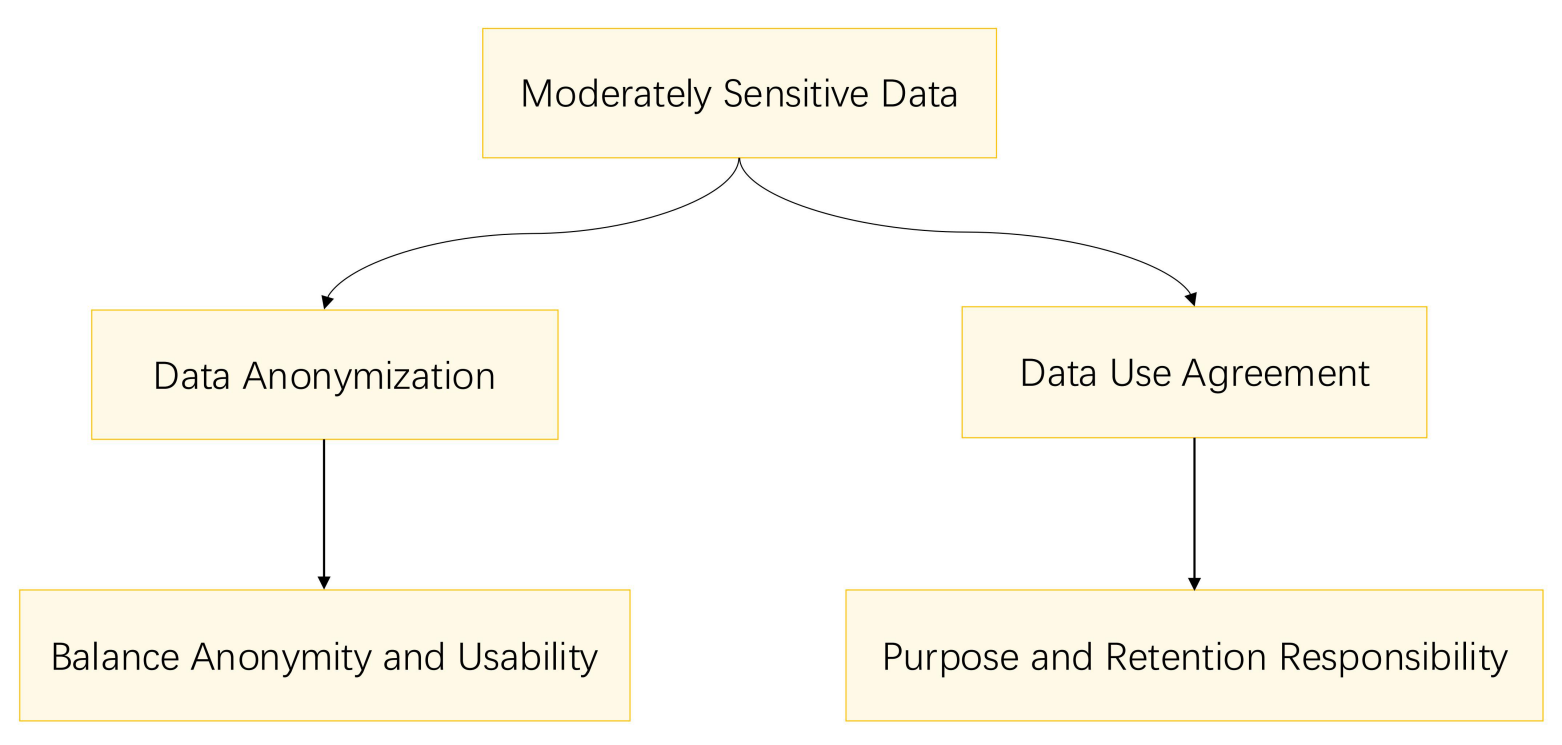
**Flowchart for moderately sensitive data privacy protection**.

While moderately sensitive data are less sensitive than highly sensitive data, they still necessitate proper data management measures to safeguard individual privacy. Fig. 4 outlines the privacy protection methods for moderately sensitive data.

#### 5.2.1 Data Anonymization

The anonymization of moderately sensitive data entails the removal or encryption of personal identifiers to prevent the data from being linked back to specific individuals. This process involves the use of technical methods to eliminate direct identifiers such as names, addresses, and telephone numbers, as well as quasi-identifiers that could potentially reveal an individual’s identity when combined with other information. As outlined in the Personal Information Protection Law of the People’s Republic of China, the determination of “sensitive personal information” is based on the likelihood of causing infringement or harm. It is crucial to maintain a balance between data anonymity, consistency, and usability during the anonymization process to prevent over-anonymization, which could diminish the research value. Reversible anonymization techniques may be employed when necessary to allow for the re-identification of individuals for data correction or legal purposes [53].

Data quality is pivotal to the accuracy and reliability of research outcomes. Consequently, robust data quality control mechanisms should be established to clean, calibrate, and standardize data. This includes employing technical means to ensure data accuracy and consistency, as well as providing adequate descriptions and documentation for data that is publicly shared or used, enabling users to understand and utilize the data correctly. For instance, Microsoft advises the use of secure administrative workstations to safeguard sensitive accounts, tasks, and data, ensuring endpoint protection.

#### 5.2.2 Data Use Agreements

Data use agreements are essential contracts that govern the exchange of specific datasets between parties. They define the permitted uses, the duration of use, and the responsibilities for data retention, among other terms. These agreements also outline the confidentiality obligations that data users must adhere to, ensuring that the data is used responsibly and within the agreed-upon parameters. In collaborative projects involving multiple organizations or researchers, multiparty data use agreements are particularly crucial to clarify the rights and obligations of each participant. Reference to the Data Use Agreement (DUA) used by the Stanford Office of Research Administration is apt here [54]; a DUA is a contract that specifies who can use and receive a unique dataset and the extent to which the data can be used and disclosed by the recipient. The DUA also assigns appropriate responsibilities for the use of the data by the researcher and the recipient, which is vital for maintaining data integrity and compliance with legal and ethical standards.

### 5.3 Minimally Sensitive Data

**Fig. 5.**
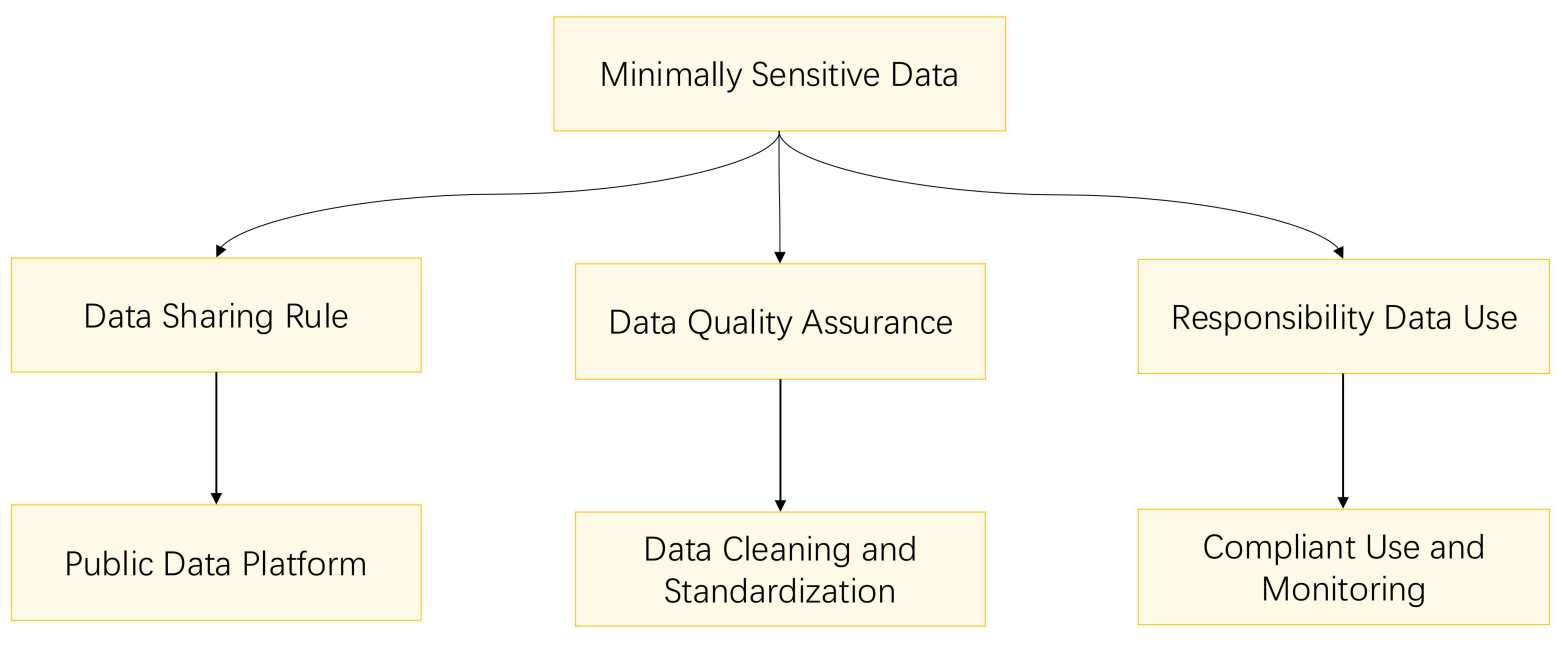
**Flowchart for minimally sensitive data privacy protection**.

Minimally sensitive data, while not containing specific personal information, must still adhere to established rules and restrictions regarding disclosure and sharing. Fig. 5 outlines the privacy protection methods for minimally sensitive data.

#### 5.3.1 Rules and Restrictions on Data Disclosure and Sharing

For minimally sensitive data, adherence to specific guidelines on disclosure and sharing is essential. These guidelines should outline the scope of data disclosure, the methods of sharing, and the formats in which data can be presented. It is recommended to establish a public data platform or data warehouse to facilitate access and use by researchers, especially when the data serves the public interest.

#### 5.3.2 Data Quality Assurance

Ensuring data quality is paramount, particularly for minimally sensitive data that is intended for public and shared use. A robust data quality control mechanism must be established to clean, calibrate, and standardize data, thereby guaranteeing its accuracy and consistency. This process encompasses various data cleaning techniques, such as the removal of duplicates, correction of errors, and resolution of inconsistencies, which are crucial for maintaining data integrity. Calibration involves the application of standard protocols to ensure comparability across data collected from diverse sources, a key aspect of reliable data analysis. Standardization refers to the conversion of data into a common format, facilitating easier analysis and interpretation. For data that is accessible and shared publicly, it is imperative to provide comprehensive data description and documentation, enabling users to understand and utilize the data effectively.

#### 5.3.3 Responsibility for Data Use

It is essential to clearly define the responsibilities and obligations of data users, which encompass adherence to data-use agreements, safeguarding data privacy, and the judicious utilization of data. A mechanism should be established for monitoring data usage and conducting regular assessments of data user behavior to ensure compliance with data policies. When executing privacy protection measures, it is advisable to implement and manage access permissions alongside robust audit and monitoring mechanisms. This process necessitates not only the engagement of hospital ethics committees but also the active involvement of the data subjects themselves. The inclusion of ethics committees in the data management process can markedly strengthen privacy protection. Furthermore, involving data subjects in the data management process can lead to more effective privacy protection outcomes.

It is crucial to note that there are three categories of data that should not be openly accessible in psychological and behavioral research. The first category involves data containing identifiable information about specific individuals, which is inherently linked to their privacy interests. For instance, personal identification information—such as names, ID numbers, and contact details—and health records are considered highly sensitive. Misuse or leakage of this information could result in severe consequences for individuals, including identity theft, fraud, and privacy violations. Consequently, such data must be stringently safeguarded and accessed or utilized only with legal permission and the individual’s explicit consent. Medical records and health information, which encompass an individual’s medical history, disease diagnoses, and treatment plans, are also classified as sensitive personal data. The unauthorized disclosure of these data could lead to personal privacy infringements or even enable improper uses like discrimination or spam marketing.

The second category comprises data related to national security. As national security is a core interest of a country, encompassing areas such as national defense, diplomacy, and intelligence, any leakage of such data could jeopardize the nation’s stability and security. Consequently, data pertaining to national security must be safeguarded with the utmost rigor to prevent unauthorized access or misuse by external entities.

Additionally, there are types of data that are afforded special protection under the law, including intellectual property rights and personal privacy. These categories of data demand stringent protection measures to ensure their security and maintain the legitimacy of their use.

## Availability of Data and Materials

Research data supporting this publication are available from the NN repository at located at https://www.x-mol.com/groups/pbicr. This narrative review and consensus recommendations are classified as a qualitative study with no available data. The other datasets used and/or analyzed during the current study are available from the corresponding author on reasonable request.
